# HMGB1 Mediates Autophagy Dysfunction via Perturbing Beclin1-Vps34 Complex in Dopaminergic Cell Model

**DOI:** 10.3389/fnmol.2017.00013

**Published:** 2017-01-31

**Authors:** Jinsha Huang, Jiaolong Yang, Yan Shen, Haiyang Jiang, Chao Han, Guoxin Zhang, Ling Liu, Xiaoyun Xu, Jie Li, Zhicheng Lin, Nian Xiong, Zhentao Zhang, Jing Xiong, Tao Wang

**Affiliations:** ^1^Department of Neurology, Union Hospital, Tongji Medical College, Huazhong University of Science and TechnologyWuhan, China; ^2^Department of Neurology, Renmin Hospital, Hubei University of MedicineShiyan, China; ^3^Department of Psychiatry, Division of Alcohol and Drug Abuse, and Mailman Neuroscience Research Center, McLean Hospital, Harvard Medical SchoolBelmont, MA, USA; ^4^Department of Neurology, Renmin Hospital of Wuhan UniversityWuhan, China

**Keywords:** HMGB1, autophagy, α-synuclein, Beclin1, Vps34, Parkinson’s disease

## Abstract

Parkinson’s disease (PD), a progressive neurodegenerative disorder, is characterized by irreversible dopaminergic neuron loss and intra-neuronal α-synuclein aggregation. High mobility group box 1 (HMGB1) has been proven to be involved in autophagy dysfunction induced by α-synuclein accumulation, and the Beclin1-vacuolar protein sorting 34 (Vps34) complex is of great importance to the initiation of autophagy. Nevertheless, the concrete interaction mechanism between HMGB1, α-synuclein and autophagy remains elusive, especially in the context of PD. Here in this study, we investigated the interaction between HMGB1 and α-synuclein in rotenone-induced PD cell models and their roles in autophagy flux. Results revealed elevated expression and cytosolic translocation of endogenous HMGB1 upon rotenone exposure. Besides, HMGB1 was found to be able to co-localize and interact with α-synuclein. Moreover, it had also been proven that HMGB1 could aggravate α-synuclein aggregation induced autophagy dysfunction via perturbing Beclin1-Vps34 complex formation. Based on these findings, we propose that HMGB1 is involved in rotenone-induced dopaminergic cell death via interacting with α-synuclein, perturbing the autophagy process, aggravating protein aggregation and finally propelling dopaminergic neurons to move from morbidity to mortality.

## Introduction

Parkinson’s disease (PD) is a progressive neurodegenerative disorder characterized by the selective degeneration of nigro-striatal dopaminergic system and the Lewy body (LB) formation in remnant dopaminergic neurons. The existing evidences suggest that aging, gene and environmental toxins may collectively contribute to the pathogenesis and progression of PD (Dawson and Dawson, 2003; Di Monte, 2003; Warner and Schapira, 2003), revealing multiple pathogenic mechanisms such as autophagy dysfunction (Pan et al., 2008), oxidative stress (Jenner, 2003), neuroinflammation (Phani et al., 2012), etc. Currently, the research focus of PD is mainly centered on the aberrant aggregation and propagation of pathogenic protein, especially in relation to α-synuclein, a major component of LBs. Moreover, blockade of the LBs formation and dissemination have been proven to be able to partly alleviate or even reverse the pathogenesis and progression of PD (Mueller et al., 2005; Müller et al., 2013; Kalia and Kalia, 2015). In particular, autophagy, an important cellular protein and organelle degradation pathway, plays an important role in blocking α-synuclein aggregation and dissemination (Xiong et al., 2012; Xilouri et al., 2016). Hence, it can be presumed that autophagy dysfunction could lead to intra-neuronal aggregation and extracellular propagation of α-synuclein, thus debilitating the dopaminergic neurons and aggravating the Parkinsonian symptoms.

High-mobility group box 1 (HMGB1), an evolutionarily conserved DNA-binding protein, has been demonstrated to be involved in several cellular processes including autophagy, apoptosis, necrosis and inflammation (Gauley and Pisetsky, 2009; Gao et al., 2011; Kang et al., 2011; Weber et al., 2015; Yu et al., 2015). Recently published reports demonstrate that cytosolic translocated HMGB1 could serve as an important cellular signal to regulate cellular metabolism and may associate with many diseases, such as cancer, ischemic stroke, neuropathic pain and even neurodegenerative diseases (Klune et al., 2008; Sims et al., 2010; Fang et al., 2012; Kang et al., 2014). In the context of PD, HMGB1 is not only found to co-localize with α-synuclein filaments in brain autopsy (Lindersson et al., 2004) but also found elevated in cerebrospinal fluid and serum (Santoro et al., 2016). Additionally, systemic administration of neutralizing antibodies to HMGB1 has also been proven to inhibit the microglial activation, suppress secondary neuroinflammation and thus prevent the dopaminergic cell death upon neurotoxin exposure in PD models (Santoro et al., 2016; Sasaki et al., 2016). In conclusion, the evidences above collectively indicate that HMGB1 may serve as a pivotal role in the pathogenesis of PD.

Beclin1 (Atg6) is an evolutionarily conserved protein family that has been shown to function in autophagy process in a wide variety of species (Furuya et al., 2005). In mammalian cells, Beclin1 function as a vacuolar protein sorting (Vps) protein and can bind to Class III PI3K Kinase (Vps34), thus forming a Beclin1-Vps34 complex which is of critical significance in autophagy modulation (Funderburk et al., 2010). What is interesting is that Beclin1 was originally identified not as an autophagy protein but rather as an interaction partner for B-cell lymphoma 2 (Bcl-2), an anti-apoptotic protein (Liang et al., 1998). Coincidentally, HMGB1, a novel endogenous Beclin1 binding protein, was proven to compete with Bcl-2 to orient Beclin1 to autophagosome, thus participating in the modulation of autophagosome formation (Kang et al., 2010). Therefore, given the study findings above, it can be presumed that HMGB1 and Beclin1-Vps34 complex may probably play an important role in autophagy modulation in the context of PD.

Here in this study, we primarily detected HMGB1 and α-synuclein expression upon rotenone exposure, discovering that HMGB1 could translocate into cytosol to co-localize with α-synuclein. To further explore whether HMGB1 can interact with α-synuclein, GFP-HMGB1 and HMGB1-small interfering RNA (siRNA) are transiently expressed in SH-SY5Y cells with or without rotenone exposure. Moreover, the interaction between HMGB1 and Beclin1-Vps34 complex is investigated as well in the case of HMGB1 overexpression and suppression, respectively.

## Materials and Methods

### Cell Culture

Human neuroblastoma cells (SH-SY5Y) were purchased from Type Culture Collection of Chinese Academy of Sciences. SH-SY5Y cells were cultured in DMEM-F12 medium (HyClone, USA) supplemented with 10% heat-inactivated new-born calf serum (Gibco, USA) in a humidified incubator with 5% CO_2_ and 95% fresh air.

### Western Blot

Total proteins were extracted from the cell lysates using RIPA lysis buffer (Cell Signaling Technology, #9806) supplemented with protease and phosphatase inhibitors (Cell Signaling Technology, #5872). Protein concentrations of the extracts were measured with BCA assay kit (Cell Signaling Technology, #7780). Afterwards, supernatant was boiled with 5× SDS loading buffer (Boster, China) for 10 min and provisionally stored at −80°C. Approximately 60 μg proteins were loaded onto SDS-PAGE, then electrophoretically transferred to PVDF (PerkinElmer, USA) membrane and blocked with 5% milk dissolved in Tris-buffered saline containing 0.1% Tween 20 (TBST) for 1 h at room temperature. The membranes were then blocked with 5% defatted milk at room temperature for 1 h and probed overnight at 4°C with the following primary antibodies: anti-HMGB1 antibody (ab79823; Abcam, Cambridge, MA, USA; 1:1000 dilution); anti-α-synuclein antibody (ab51253; Abcam, Cambridge, MA, USA; 1:500 dilution); anti-Beclin1 antibody (ab51031; Abcam, Cambridge, MA, USA; 1:1000 dilution) and anti-p62 antibody (Cell Signaling technology, NO.8025S; 1:1000 dilution); anti-LC3A/B antibody (Cell Signaling technology, NO.12741S; 1:1000 dilution); and anti-β-actin (ACTB) antibody (CW0281A; Comwin Biotech, USA; 1:3000 dilution). After incubation with horseradish peroxidase-conjugated secondary antibodies for 1 h at room temperature, the signals were detected by Kodak Professional Film Developer. Relative band intensities were quantified using Quantity One software.

### Co-Immunoprecipitation Analysis

SH-SY5Y cells were lysed at 4°C in ice-cold immunoprecipitation (IP) assay lysis buffer (Beyotime, China; P0013J) for 10 min, then the cells lysates were centrifuged for 3 min at 12,000 g. Concentrations of proteins in the supernatant were determined by bicinchoninic acid assay (Abcam, UK; ab207003). Then the extracted proteins were divided into two parts, one contained whole cell lysis (WCL) which would be assessed by Western blot (WB) directly and another prepared for co-immunoprecipitation (Co-IP). Besides, a negative control group was established, which contained normal cell lysates and would be incubated by IgG in Co-IP. Before Co-IP, the samples containing equal amounts of proteins were pre-cleared with protein A or protein G agarose/sepharose (Cell Signaling Technology, #9863) at 4°C for 3 h and subsequently incubated with irrelevant IgG or antibodies (2–5 μg/ml) in the presence of protein A or G agarose/sepharose beads (Cell Signaling Technology, #9863) for 2 h at room temperature or overnight at 4°C with gentle shaking. After incubation, agarose/sepharose beads were extensively washed with PBS, and proteins were eluted by boiling in 2× SDS sample buffer before SDS-PAGE electrophoresis.

### Gene Transfection

Plasmid encoding HMGB1 fused with GFP was provided by Dr. George Hoppe at University of Pittsburgh and Dr. Marco Bianchi at San Raffaele University. Control DNA poly β siRNA and siRNA were purchased from Guangzhou RiboBio Co. Ltd, China, and were operated according to standard transfection protocols for cell cultures. GFP-HMGB1 or siRNA-HMGB1 was transfected into SH-SY5Y cells using Lipofectamine 2000 reagent (Invitrogen, Waltham, MA, USA) according to the manufacturer’s instructions. In accordance with the experimental protocols, the cells were prepared for subsequent treatments 48 h after the transfection procedures.

### Immunofluorescence

SH-SY5Y cells were seeded on 12-well plates and fixed in 4% paraformaldehyde for 10 min before detergent extraction with 0.1% Triton X-100 for 15 min at room temperature. The wells were saturated with 1% bovine serum albumin in phosphate-buffered saline (PBS) for 1 h at room temperature and processed for immunofluorescence with anti-HMGB1 antibodies followed by Fluorescein Isothiocyanate (FITC)-conjugated anti-rabbit IgG. Nuclear morphology was analyzed after staining with the fluorescent dye Hoechst 33342 (Sigma-Aldrich, USA) for 10 min. Cells were washed with PBS three times for 5 min each between incubation steps. Images were taken under a confocal microscope.

### Transmission Electron Microscopy

Prepared cell samples were quickly rinsed in PBS 3× and fixed with a 0.1 mol/L phosphate buffer solution (pH 7.4) containing 3% glutaraldehyde plus 2% paraformaldehyde. Next, the cell samples were fixed by 1% OsO_4_ for 1 h to produce osmium black before dehydration. Sections of the cells were prepared with a diamond knife on a Reichert-Jung Ultracut-E ultra-microtome. Then the cell samples were stained with UrAc (20 min) followed by 0.2% lead citrate (2.5 min) for observation under a JEM 1011CX electron microscope (JEOL USA, Inc.). Digital images were acquired by the Advanced Microscopy Techniques imaging system.

### Cell Morphology and Cell Apoptosis Evaluation

SH-SY5Y cells were cultured in 6-well plates and treated with the indicated drug combination for 24 h after transfecting with/without HMGB1 plasmid or siRNA. The cell morphology was observed under an inverted microscope (IX70, Olympus, Japan) and recorded using a CMOS camera (Infinity Lite, USA). For Hoechst staining, cells cultured on coverslips in 12-well plates were fixed in 4% paraformaldehyde for 30 min after predesigned treatments. Hoechst 33258 (Sigma, USA) was used to stain the cell nuclei after PBS washing for 3× and incubated for 10 min before observing. The positively stained cells with nuclear deformities were counted in 10 random fields under the confocal microscope. For flow cytometry analysis, cells were washed three times with PBS after harvest and stained with Annexin V-FITC and propidium iodide (PI) according to the manufacturer’s instructions. The cells were then washed and analyzed with a FACScan flow cytometry (Becton Dickinson, Franklin Lakes, NJ, USA).

### Statistical Analysis

All statistical analyses were performed using SPSS 20.0 (SPSS, Chicago, IL, USA). Data are expressed as mean ± standard error (SEM). Differences among groups were analyzed by one-way analysis of variance (ANOVA) followed by Least square difference’s *post hoc* test. *P* < 0.05 was considered significantly different. All the data were obtained from three independent experiments.

## Results

### Elevated HMGB1 and α-Synuclein Expression Upon Rotenone Exposure

**Figure 1:**
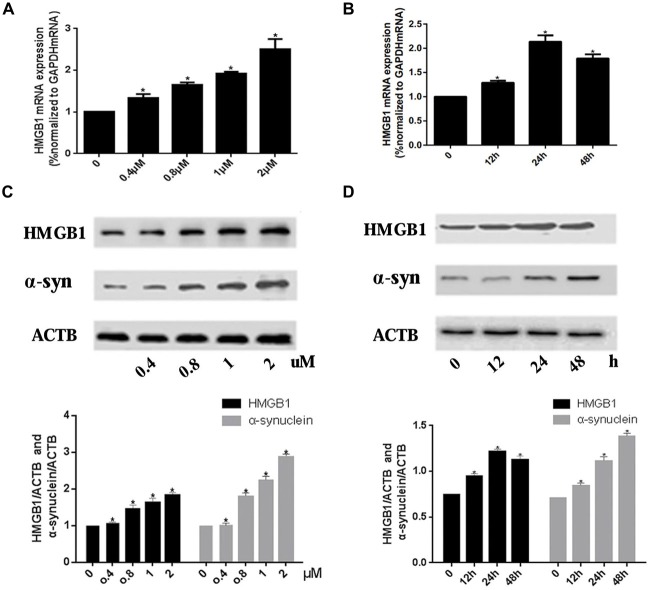
**High mobility group box 1 (HMGB1) and α-synuclein overexpressed upon rotenone exposure.** SH-SY5Y cells were treated either with rotenone concentration increment (0, 0.4 μM, 0.8 μM, 1 μM, 2 μM) pattern **(A)** or with time stretch pattern (0 h, 12 h, 24 h or 48 h) exposed to 1 μM rotenone abidingly **(B).** The mRNA levels of HMGB1 were measured by RT-PCR in **(A,B)**, while the protein expression level of HMGB1 and α-synuclein were determined by Western blot (WB; **C,D**). Relative intensity is normalized to that of β-actin (ACTB; **C,D**). Data are presented as the mean ± standard error (SEM) from three independent experiments. *P < 0.05.

Real-time PCR and WB were used to investigate the expression level of HMGB1 and α-synuclein upon rotenone exposure. Results showed that the HMGB1 expression (both at mRNA and protein level) was elevated in a concentration dependent manner. Moreover, HMGB1 protein expression pattern detected by WB was consistent with that of HMGB1 mRNA (Figures 1A,C). While in the time course of HMGB1 mRNA expression, with persistent 1 uM rotenone incubation, it revealed a constant increase which peaked at 24 h and then slightly decreased at 48 h (Figure 1B). In contrast, α-synuclein expression demonstrated a time and concentration dependent manner under rotenone treatment (Figures 1C,D).

### HMGB1 Presented Cytosolic Translocation and Interaction With α-Synuclein Upon Rotenone Exposure

**Figure 2:**
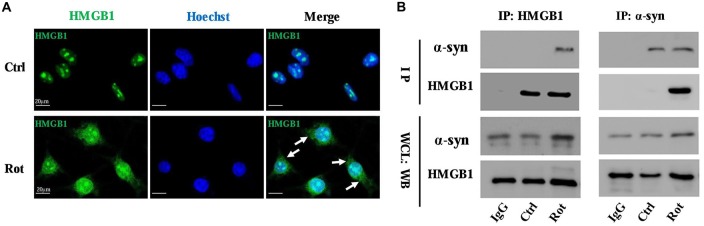
**HMGB1 translocated to cytoplasm and interacted with α-synuclein.** The overall HMGB1 expression increased under rotenone treatment (1 μM, 24 h), accompanying cytoplasmic translocation (marked by arrows) as proven by confocal microscopy **(A)**. The interaction between HMGB1 and α-synuclein were further confirmed by co-immunoprecipitation (Co-IP), which indicated that HMGB1 and α-synuclein could form co-precipitation complex upon rotenone exposure **(B)**. WCL, whole cell lysate; Rot, rotenone; Ctrl, control; IP, immunoprecipitation; WB, Western blot.

The interaction between HMGB1 and α-synuclein was primarily investigated by laser confocal microscopy and then confirmed by Co-IP. The laser confocal microscopy indicated that HMGB1 was mainly located in the nucleus and scarcely existed in the cytoplasm in control SH-SY5Y cells (Figure 2A). Nevertheless, after 1 uM rotenone incubation for 24 h, HMGB1 expression levels were markedly elevated, with some shuttling from the nucleus to the cytoplasm (Figure 2A). Besides, the HMGB1 distribution pattern has changed as well, transforming from a granularly concentrated pattern to a muddily dispersive one (Figure 2A). Moreover, when compared with control group, HMGB1 and α-synuclein had been confirmed to form co-precipitation complex after rotenone exposure as proven by Co-IP (Figure 2B). In conclusion, the outcomes above indicated that HMGB1 could present overexpression, cytosolic translocation and interaction with α-synuclein upon rotenone exposure.

### HMGB1 Regulated α-Synuclein Expression

**Figure 3:**
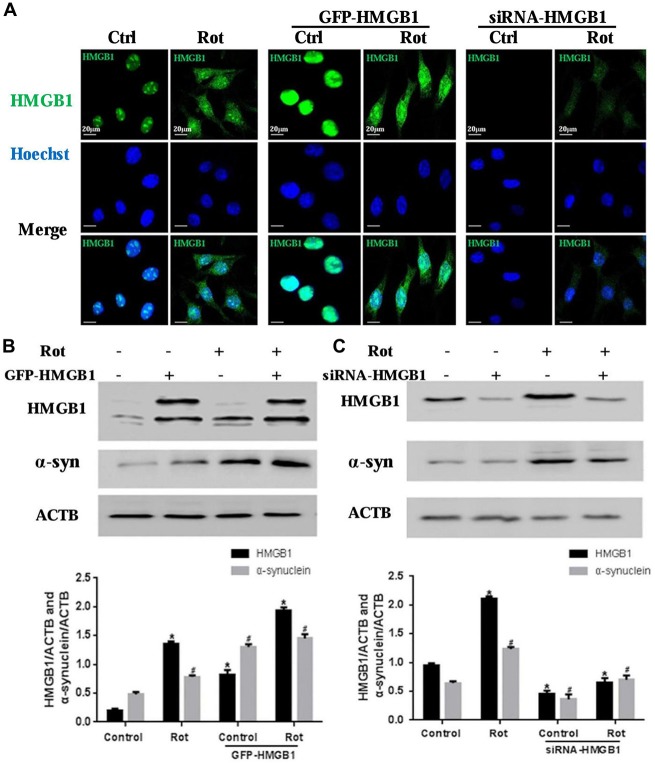
**HMGB1 regulated α-synuclein expression.** SHSY-5Y cells were transfected with GFP-HMGB1 plasmid and small interfering RNA (siRNA)-HMGB1 respectively. Confocal microscopy was used to detect HMGB1 expression and subcellular location after rotenone treatment (1 μM, 24 h) **(A)**. The expression level of HMGB1 and α-synuclein were determined by WB after GFP-HMGB1 plasmids **(B)** or siRNA-HMGB1 **(C)** transfection. Relative intensity is normalized to that of ACTB/β-actin **(B,C)**. Data are presented as the mean ± SEM from three independent experiments. *^#^*P* < 0.05.

To further explore the relationship between HMGB1 and α-synuclein, wild-type HMGB1-GFP fusion protein and HMGB1-siRNA were transiently expressed in SH-SY5Y cells with or without rotenone exposure (Figure 3A). What is interesting is that HMGB1 expression level, compared with respective control group, were significantly elevated upon rotenone exposure, no matter the upward or downward manipulation of HMGB1. Moreover, the HMGB1 distribution patterns demonstrated similar changes to that identified in Figure 2A under rotenone treatment, regardless of the HMGB1 manipulation approaches (Figure 3A). The expression level of HMGB1 had also been proven to coincide with that of α-synuclein both in the context of HMGB1 manipulation and rotenone exposure. In particular, HMGB1 overexpression could increase α-synuclein expression and the positive effect could be further intensified by rotenone incubation (Figure 3B). While in the context of HMGB1 suppression, it was found that α-synuclein expression decreased as well, but partly restored by rotenone exposure (Figure 3C). Therefore, the study results above indicated that HMGB1 might regulate the expression of α-synuclein, while rotenone exposure could exert a positively intensifying effect.

### HMGB1 Mediated Autophagy Impairment via Perturbing Beclin1-Vps34 Complex Formation

**Figure 4:**
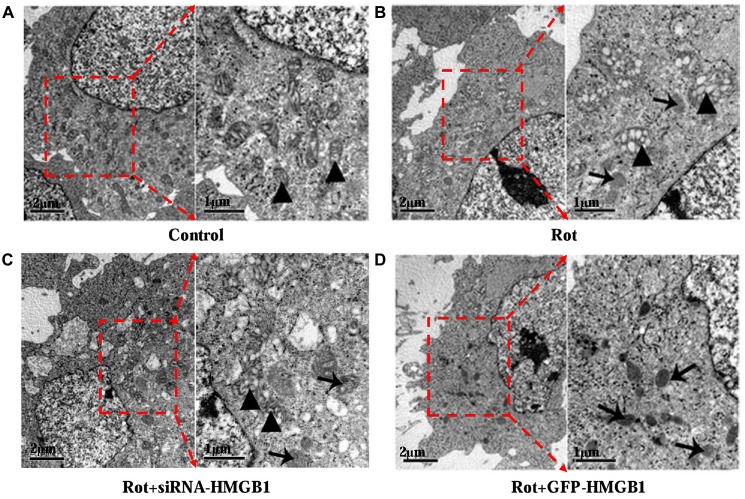
**The ultrastructural view of HMGB1 mediated autophagy dysfunction.** In control group, SH-SY5Y cells presented normal cellular ultrastructure and mitochondria (**A**, marked by triangles). In comparison, autophagosomes (marked by arrows) could be observed in Rot group (incubation with rotenone for 24 h), accompanying deformed mitochondria and swelling mitochondrial cristae (**B**, marked by triangles). When transfected with siRNA-HMGB1, the mitochondrial deformation in Rot + siRNA-HMGB1 group (incubation with rotenone for 24 h after siRNA-HMGB1 transfection) was partly alleviated (**C**, marked by triangles), but still presenting sporadic autophagosomes (marked by arrows). By contrast, the GFP-HMGB1 group (incubation with rotenone for 24 h after GFP-HMGB1 transfection) revealed severely deformed cellular ultrastructure and numerous autophagosomes (**D**, marked by arrows).

Given that HMGB1 could modulate α-synuclein expression and abnormal α-synuclein aggregates were usually degraded by autophagy-lysosome pathway, whether HMGB1 could exert a modulatory effect on autophagy was thereby investigated. In comparison with control group (Figure 4A), after incubating SH-SY5Y cells with 1 μM rotenone for 24 h, it revealed autophagosome formation and mitochondrial deformation under transmission electron microscope (Figure 4B), which could be further aggravated by HMGB1 overexpression (Figure 4C). Nevertheless, the mitochondrial deformation in SH-SY5Y cells could be partly alleviated when transfected with siRNA-HMGB1, but still accompanying sporadic autophagosomes (Figure 4D).

**Figure 5:**
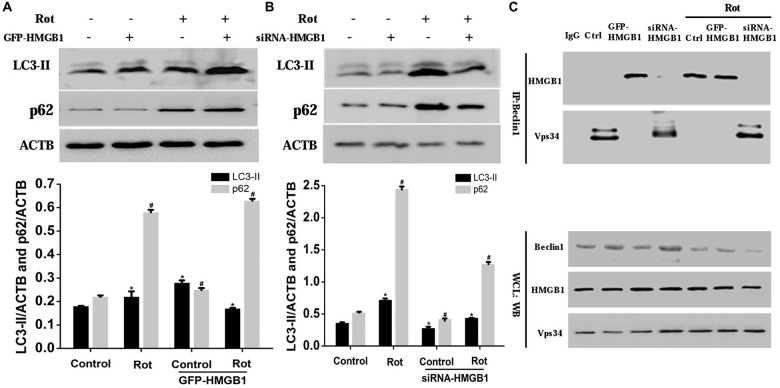
**HMGB1 mediated cellular autophagy dysfunction via Beclin1-vacuolar protein sorting 34 (Vps34) complex perturbation.** SH-SY5Y cells were incubated with 1 uM rotenone for 24 h after GFP-HMGB1 plasmids **(A)** or siRNA-HMGB1 **(B)** transfection. The expression of light chain 3 (LC3) and P62 in different groups was determined by WB **(A,B)**. HMGB1, in the context of overexpression or rotenone treatment, could interact with Beclin1 and thereby inhibited the Beclin1-Vps34 complex formation, while HMGB1 suppression could partly reverse the autophagy dysfunction **(C)**. *^#^*P* < 0.05.

To confirm the effect of HMGB1 on autophagy, we explored the expression changes of autophagy associated proteins including microtubule-associated protein light chain 3-II (LC3-II) and p62 upon HMGB1 manipulation (Figures 5A,B). As a result, we found that HMGB1 overexpression could remarkably augment autophagy marker protein (LC3-II and p62) expression no matter with or without rotenone pretreatment (Figure 5A). In contrast, inhibition of HMGB1 expression by siRNA could obviously reduce LC3-II and p62 expression, but partly restored by rotenone incubation (1 μM, 24 h; Figure 5B). In general, the expression level of LC-I, LC-II and p62 signified the feasibility of autophagy flux. When revealing more conversion of LC-I to LC-II and less p62 expression, it indicated feasibility of the autophagy flux; while the more conversion of LC-I to LC-II and elevated p62 expression implied normal initiation but not sustaining of autophagy flux. In conclusion, the study outcomes above indicated that HMGB1 could initiate but not sustain the autophagy flux.

To further clarify how HMGB1 modulated the autophagy flux and whether Beclin1 or Vps34 was involved in this process, we transiently transfected SH-SY5Y cells with GFP-HMGB1 or siRNA-HMGB1 and performed Co-IP to explore potential interaction. As a result, it revealed that endogenous Beclin1 normally bind to Vps34 but not HMGB1, as shown by Co-IP in control group (Figure 5C). However, after rotenone treatment, Beclin1 turned to bind to HMGB1 to form HMGB1-Beclin1 complex. Considering that HMGB1 expression could be upregulated upon rotenone treatment (Figures 1–3), it could be presumed that HMGB1 could disintegrate the Beclin1-Vps34 complex (Figure 5C). Moreover, HMGB1 overexpression maintained the HMGB1-Beclin1 interaction no matter rotenone was added or not. In contrast, when cells were transfected with siRNA to reduce HMGB1 expression, the interaction between Vps34 and Beclin1 in cytoplasm would restore (Figure 5C). Hence, these results suggested that HMGB1 had a higher affinity for Beclin1 and thereby suppress autophagy process by blocking the Beclin1-Vps34 complex formation, all of which could be positively intensified by rotenone exposure.

### HMGB1 Influenced Cellular Morphology and Viability

**Figure 6:**
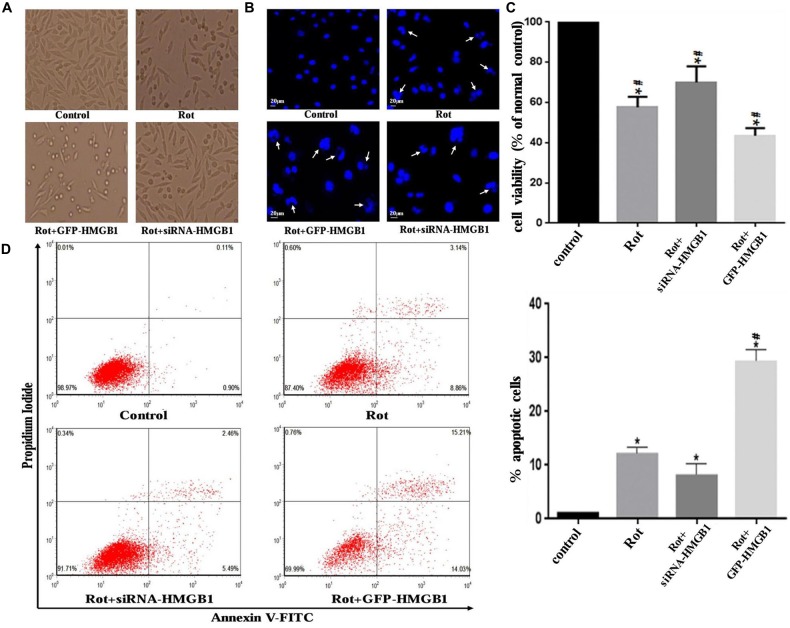
**HMGB1 influenced cellular morphology and viability.** Cellular morphology was observed under a phase-contrast microscope (×200 magnifications) when treated with 1 μM rotenone after transfection with GFP-HMGB1 plasmids or siRNA-HMGB1 **(A)**. SH-SY5Y cells were stained with Hoechst 33258 to observe the nuclear structure alterations (**B**, marked by arrows). The cellular viability and apoptosis rate were determined by Hoechst 33258 staining **(C)** and flow cytometry **(D)** in different groups, indicating that GFP-HMGB1 transfection could exacerbate rotenone induced cell death while HMGB1 suppression could partly alleviate it. *P < 0.05 compared with control group; ^#^*P* < 0.05 compared with the other groups.

Morphological changes in SH-SY5Y cells were assessed by phase-contrast microscope, which indicated that HMGB1 overexpression or rotenone exposure could contribute to cellular shrinkage and nuclear structure destruction (Figures 6A,B). Hoechst 33258 staining demonstrated nuclear fragmentation, karyopyknosis, karyolysis and chromatin margination (Figure 6B, marked by arrows), which could be used to indirectly indicate cell viability (Figure 6C). Moreover, GFP-HMGB1 plasmids transfection further aggravated the cellular morphology and nuclear structure damages. Nevertheless, HMGB1 suppression could partly alleviate these phenomena (Figures 6A–C). The cell apoptosis rate was determined by flow cytometry. Data showed that rotenone treatment could induce an apoptosis rate of 12.00 ± 0.23% and HMGB1 overexpression resulted in a higher apoptosis rate of 29.24 ± 0.21%, while HMGB1 suppression pre-treatment (siRNA-HMGB1 transfection) only result in an apoptosis rate of 7.95 ± 0.14% (Figure 6D).

## Discussion

HMGB1, a non-histone DNA binding protein, is capable of promoting DNA restoration, enhancing transcription in the nucleus and regulating cell differentiation, inflammation, apoptosis, autophagy and cell cycle in the cytoplasm (Livesey et al., 2012; Yang et al., 2013). Besides, HMGB1 is proven to be involved in several neurodegenerative disorders, such as amyotrophic lateral sclerosis (ALS; Lo Coco et al., 2007; Hwang et al., 2013), Alzheimer’s disease (AD; Takata et al., 2003, 2012; Jang et al., 2013), Huntington’s disease (Okazawa, 2011; Min et al., 2013) and PD (Lindersson et al., 2004; Song et al., 2014; Santoro et al., 2016; Sasaki et al., 2016). For example, HMGB1 was demonstrated to promote disease progression by aggravating neuroinflammation and selectively damaging motor neurons in a mouse model of ALS (Lo Coco et al., 2007). In addition, a more recent study showed that systemic administration of neutralizing antibodies to HMGB1 could relieve dopaminergic neuron death upon neurotoxin exposure via inhibition of microglial activation and subsequent neuroinflammation in PD models (Santoro et al., 2016; Sasaki et al., 2016), thus implying the involvement of HMGB1-modulated neuroinflammation dysregulation in the pathogenesis of PD. While in the context of AD, HMGB1 was proven to be involved in the accumulation of amyloid-β-containing neuritic plaques which was, as a matter of fact, caused by impaired autophagy process (Takata et al., 2003). Also, it had also been demonstrated that HMGB1 could serve as a ligand for α-synuclein, binding preferentially to α-synuclein aggregates or presenting in LBs just as identified in brain tissue of dementia with LBs or PD patients (Lindersson et al., 2004). Moreover, Song et al revealed that overexpressed α-synuclein could bind to HMGB1, impair its cytosolic translocation and block the HMGB1-Beclin1 interaction, ultimately resulting in autophagy inhibition (Song et al., 2014). However, whether and how HMGB1 exert a modulatory effect on autophagy still remain elusive. Herein, we intend to investigate the exact interaction mechanism between HMGB1, autophagy and α-synuclein, aiming to push the autophagy research in PD a step further.

As we all know, cellular autophagy may lead to two opposite outcomes: closely modulated autophagy can remove long-lived proteins and dysfunctional organelles and thus exert protective effects (Kang et al., 2010; Ebrahimi-Fakhari et al., 2012), while disorganized autophagy has been confirmed to induce autophagic cell death and even autosis (Liu and Levine, 2015). Therefore, it can be presumed that autophagy is a double-edged sword (Shintani and Klionsky, 2004; Tung et al., 2012), of which Beclin1 is, in fact, a vital factor to balance the autophagy-induced cytoprotective and cytotoxic effects (Erlich et al., 2006). While a study in PC12 cells revealed that intracellular promotion of either HMGB1 or Beclin1 could up-regulate α-synuclein degradation and ameliorate α-synuclein mediated autophagy inhibition (Wang et al., 2016), but the relationship between HMGB1 and Beclin1 in autophagy process still remained elusive. Moreover, the downstream effector molecules of HMGB1 and Beclin1 are unknown as well. To clear up the doubts above, we launched an in-depth investigation.

**Figure 7:**
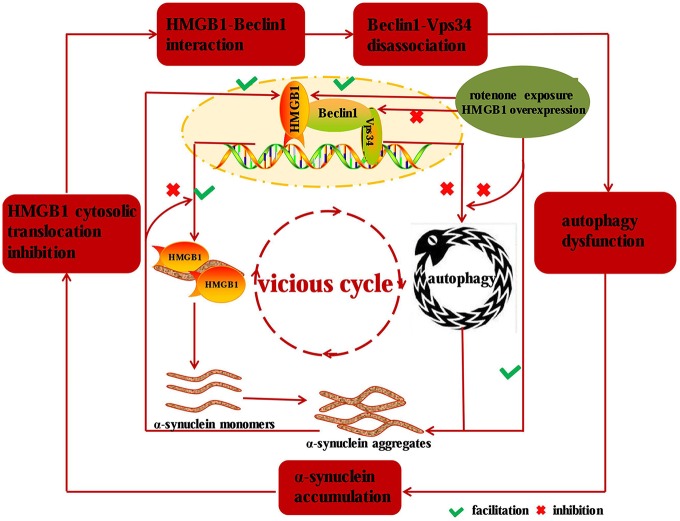
**Diagram illustrating the mechanisms implied in HMGB1 mediated autophagy impairment.** HMGB1 can bind to Beclin1 upon rotenone exposure or manipulative overexpression and thereby perturbs the Beclin1-Vps34 complex formation, which subsequently results in α-synuclein accumulation. While the accumulated α-synuclein can inhibit the cytosolic translocation and facilitate the disassociation of Beclin1-Vps34 complex, thus forming a vicious cycle to drive the dopaminergic neurons from morbidity to mortality in PD.

In our study, we found that HMGB1 and α-synuclein expression could be markedly elevated in a concentration or time dependent manner upon rotenone exposure, and the overexpressed HMGB1 could also translocate into cytosol and interact with α-synuclein. Moreover, HMGB1 could increase α-synuclein expression and the positive effect could be further intensified by rotenone incubation. Beyond that, HMGB1 overexpression had also been proven to promote the initiation but not sustaining of autophagy, as a result of more conversion of LC3-I to LC3-II and elevated p62 expression. As a result, the block of autophagy flux lead to further α-synuclein overexpression and subsequent aggregation (Mader et al., 2012; Garcia-Garcia et al., 2013), thus forming a vicious cycle to propel the condition from bad to worse. Similarly, a study had revealed the manipulatively overexpressed α-synuclein could bind to both cytosolic and nuclear HMGB1, impair its cytosolic translocation and subsequent interaction with Beclin1, which finally resulted in autophagy inhibition and further aggravated α-synuclein accumulation (Figure 7; Song et al., 2014). Nevertheless, overexpression of Beclin1 could instead restore autophagy, promote α-synuclein clearance (Song et al., 2014) and ultimately break the vicious cycle shown in Figure 7. Moreover, HMGB1 inhibitor glycyrrhizin pretreatment or HMGB1 knockdown had also been proven to facilitate the autophagy flux and rescue 3-nitropropionic acid induced striatal damage (Qi et al., 2015), which is precisely consistent with the study results. In conclusion, this study has identified that HMGB1 have a higher affinity for Beclin1 and thereby perturb the Beclin1-Vps34 complex formation, thus resulting in subsequent autophagy dysfunction and α-synuclein accumulation. Based on the study results and pre-existing conclusions, it can be further presumed that the dysfunctional autophagy induced α-synuclein accumulation can in turn exacerbate HMGB1 mediated autophagy failure to form a vicious cycle (Figure 7).

## Author Contributions

YS, JY, JH, ZL, NX, ZZ, JX, TW designed and drafted the manuscript; JY, HJ, CH, GZ, LL, XX, JL, YS conducted the experiments; JH, YS, ZL, NX, ZZ, JX, TW analyzed and supervised the experiments. All authors reviewed and approved the manuscript.

## Funding

This work was supported by grants 31171211, 81471305 and 81671260 from the National Natural Science Foundation of China (to TW), grant 81301082 from the National Natural Science Foundation of China (to JH), grant 81200983 from the National Natural Science Foundation of China (to NX), grant 2012B09 from China Medical Foundation (to NX) and grant 0203201343 from Hubei Molecular Imaging Key Laboratory (to NX). The funders had no role in study design, data collection and analysis, decision to publish or preparation of the manuscript.

## Conflict of Interest Statement

The authors declare that the research was conducted in the absence of any commercial or financial relationships that could be construed as a potential conflict of interest.
